# Exploring the latest understanding on the role of immune mediators, genetic and environmental factors in pathogenesis of allergic rhinitis: a systematic review

**DOI:** 10.3389/falgy.2023.1223427

**Published:** 2023-08-25

**Authors:** Shaimaa Albloushi, Mona Al-Ahmad

**Affiliations:** ^1^ Al-Rashed Allergy Center, Ministry of Health, Kuwait City, Kuwait; ^2^ Microbiology Department, College of Medicine, Kuwait University, Kuwait City, Kuwait

**Keywords:** allergic rhinitis, cytokines, pathogenesis, genetic factor, enviromental factors

## Abstract

**Introduction:**

Despite well-defined clinical phenotypes of chronic rhinitis, the underlying in-depth pathophysiological mechanism, particularly with reference to the involvement of immune mediators, genetic, and environmental factors, are still not fully understood. Therefore, our aim was to give updated information on the pathogenesis of allergic rhinitis (AR), with an emphasis on the role of cytokines in adults aged 18 years and above. Additionally, we investigated the impact of genetic and environmental factors in the pathogenesis of AR.

**Results:**

A search in various databases identified 1,178 records, and 18 studies were ultimately selected from January 2018 to April 2023. The total sample size in our studies was 4,317, with 2,186 in the experimental and 2,131 in control groups, respectively. The mean age was 33.4 years, with 43% were male, while 57% were female. According to the selected studies, various factors, including immune mediators, particularly cytokines, genetic, and environmental factors, were identified in the development of AR.

**Conclusion:**

The selected studies presented findings on different factors and sub-factors in the pathogenesis of AR, making it a challenge for us to compare their results. However, based on our findings, researchers can link our identified factors to potential therapies for AR.

## 1. Introduction

In order to effectively treat a disease, understanding its true underlying mechanism is essential (1). Unfortunately, for many chronic diseases, these mechanisms are not fully understood, which leads to a reliance on symptom-based management. Chronic rhinitis is one such disease (2). It refers to inflammation of the nasal mucosa that persists for a minimum of 12 weeks each year (3). Allergic rhinitis (AR) and non-allergic rhinitis (NAR) are two well-recognized subgroups of rhinitis (4).

The pathogenesis of chronic rhinitis is linked to immune mediators, environmental, genetic, and epigenetic factors. However, the available data is insufficient to draw definitive conclusions about the involvement of these factors particularly in AR (5). The available data have focused on the role of environment, genetics, and epigenetics in the pathogenesis of AR. The development of AR in individuals with atopic tendencies is believed to be strongly influenced by their exposure to various external environmental factors (6). Other than environmental factors, various genes associated with immune-related diseases, such as allergic and autoimmune disorders, are also involved (7).

The prevalence of AR varies among studies due to the lack of an acceptable definition for both conditions (8, 9). A review by Savoure et al. reported a wide range of prevalence, varying from 1% to 63% from 184 studies. The overall median prevalence rates of unspecified rhinitis, and AR, were 29.4% and 18.1%, respectively (9). The severity of chronic rhinitis, particularly AR symptoms, is associated with lower quality of life. A large Spanish cohort study concluded that AR symptoms have a considerable impact on the quality of life and sleep quality for all patients with AR (10).

However, despite well-defined clinical phenotypes of chronic rhinitis, the underlying pathophysiological mechanism, particularly with reference to the involvement of cytokines, genetic, and environmental factors, is still not fully understood (2). The significance of cytokines in AR is considered more significant among these factors. Cytokines possess roles that depend on the context and determine their usual physiological function. However, when cytokine pathways become dysregulated or chronically activated, they can disrupt the equilibrium of tissues, moving away from a state of balanced stability toward immunopathology associated with disease (11). The aim of the review was to give updated information on the pathogenesis of AR at the molecular level, with an emphasis on the role of cytokines in adults aged 18 years and above. Additionally, we investigated the impact of genetic and environmental factors in the pathogenesis of AR.

## 2. Method

On May 02, 2023, a literature search was conducted based on PRISMA (Preferred Reporting Items for Systematic Reviews and Meta-Analyses) guidelines. The search included relevant articles published between 2018 and 2023 in PubMed, Scopus, Wiley online library, and Google Scholar. Keywords such as Rhinitis, Chronic rhinitis, Allergic rhinitis, Non-Allergic Rhinitis, Pathophysiology, Etiology, Mechanism, Environmental factors, Epigenetic factors, Inflammatory Mediators, Inflammatory Biomarkers, Cytokines, Interleukin, Interferon, Chemokines, and Immune mediators were used in various combinations. The searches were conducted separately for each primary database using Boolean operators (AND) and (OR) in combination with Medical Subject Subheadings (MeSH) terms and key text words.

Our inclusion criteria include controlled trials that investigated the role of cytokines, genetic factors, and environmental factors in the pathogenesis of AR. Experimental or animal studies, non-English language studies, non-peer-reviewed studies, conference abstracts, papers, letters, and unpublished manuscripts were excluded. Likewise, studies that investigated the pathogenesis for coexistence of AR with other diseases were also excluded.

The potential studies were initially screened based on their abstracts and article titles. Subsequently, full-text articles of potential studies were evaluated by two independent researchers. Any discrepancies were discussed until a consensus was reached to ensure the accuracy of the research. We utilized descriptive statistics, such as mean and median, to obtain summary measures.

## 3. Results

### 3.1. Literature search

**Figure 1:**
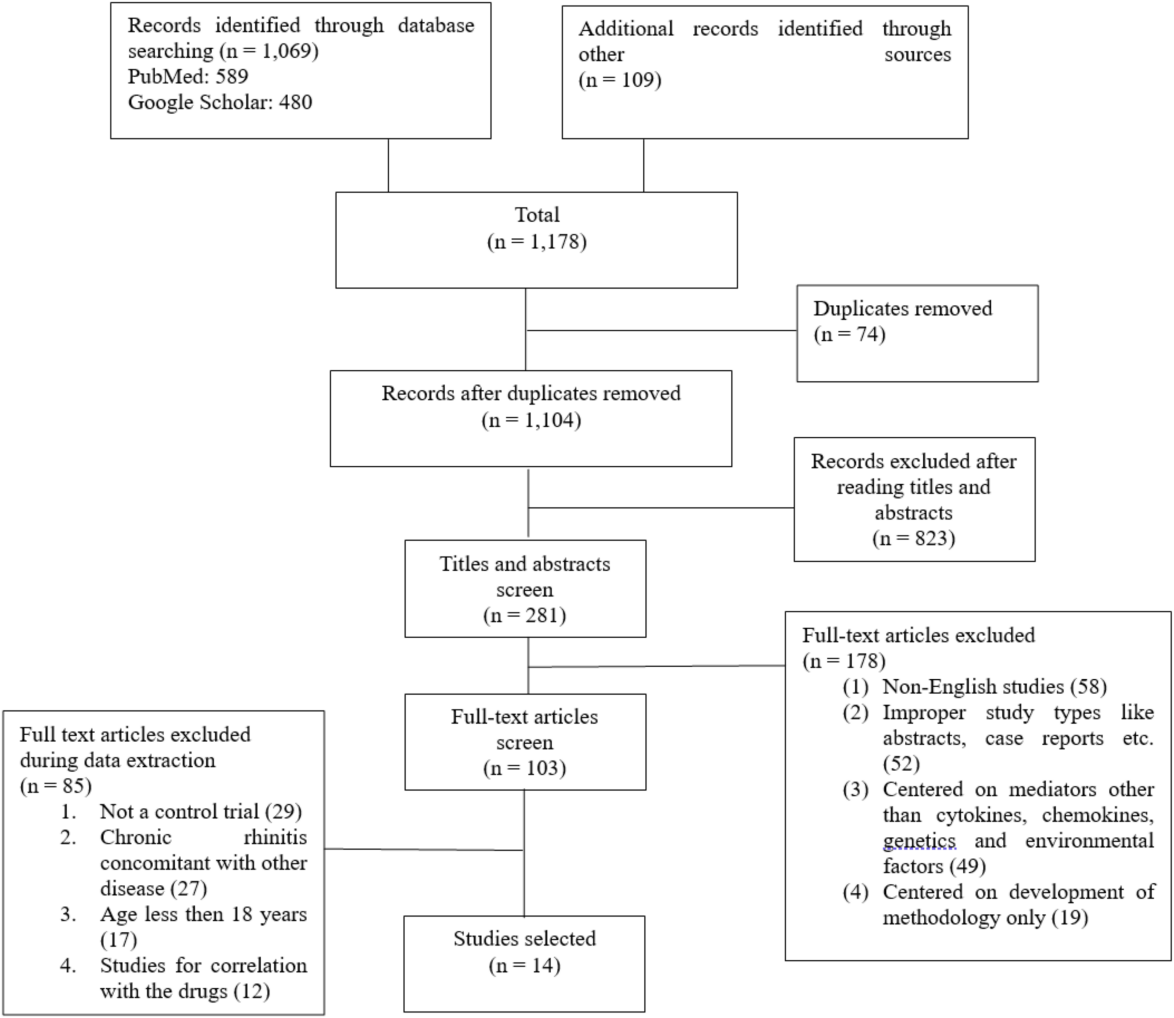
PRISMA flowchart for the searching and screening studies.

A search was conducted in databases as well as other sources like relevant journals and bibliographic searches, resulting in the identification of 1,178 records. After a screening process based on titles, abstracts, and full-text articles, 103 articles were screened. Out of the 103 articles, 85 were excluded for not meeting the inclusion criteria, such as not being a controlled trial, involving patients under 18 years of age, studied only the correlation of different factors in the pathogenesis of AR with drugs, or was centered on the pathogenesis non-allergic rhinitis or mixed rhinitis. In the end, 14 studies from January 2018 to April 2023 were ultimately selected. These studies were chosen based on their relevance to immune mediators i.e., cytokines, genetic factors, and environmental factors in the pathogenesis of AR. The PRISMA flow chart in Figure 1 illustrates the selection process of the studies.

### 3.2. Characteristic analysis of included studies

The studies selected for analysis comprised of seven conducted in China, three in Iran, and one each in Turkey, Malaysia, Romania, and Australia. Out of the 14 selected studies, four were cross-sectional, five were case-control, two were comparative studies, and three were genome-wide association studies (GWAS). The total sample size for these studies was 4,317, with 2,186 in the experimental and 2,131 in the control groups, respectively. The mean age was 33.4 years with 43% were male, while 57% were female. Various diagnostic criteria were used for the diagnosis of AR, including clinical examination, allergic rhinitis and its impact on asthma (ARIA) guidelines (12), and skin prick tests, among others. The detailed analysis of the characteristics of the selected studies are presented in Tables 1–3.

**Table 1:** Investigation of immune mediators in AR: characteristics of included studies.

Author	Country	Study design	Sample size		Sample demographics	Diagnostic criteria	Main outcome
			Exp	Con			
(13)	Romania	Prospective study	24	15	Age, 36 years; 35% male	Skin prick test	Mean serum CCL3 concentration was significantly higher in patients with AR than in controls
(14)	Iran	Cross-sectional study	37	30	Age, 35 years; 34% male	Clinical examination	AR patients had higher serum levels of RvE1 and LTB4, but a lower ratio of RvE1 and RvD1 to LTB4, IL-10 and TGF-β cytokines.
(15)	China	Comparative study	25	31	Age, 31 years; 44% male	Clinical symptoms, Nasal endoscopic examination.	AR had more CD4+ T cells and higher levels of IL-10, IL-6, and IFN-γ, but lower levels of Th1 and Th17 subsets with chemokine receptors
(16)	Iran	Cross-sectional study	35	35	Age, 31.4 years; 38.5% male	Skin prick test	No significant differences in IL-25 and IL-33 levels between fungal and non-fungal AR
(17)	Turkey	Prospective study	65	31	Age, 31.5 years; 46.5% male	History, physical examination, and laboratory findings	IL-17. IL-22, and TGF-β were higher in patients of AR, while IFN-γ was lower

AR, allergic rhinitis; ARIA, allergic rhinitis and its impact on asthma (18); ENT, ear, nose, and throat; LT, leukotriene; IL, interleukin; IFN, interferon; TGF, transforming growth factor.

**Table 2:** Investigation of genetic factors in AR: characteristics of included studies.

Author	Country	Study design	Sample size		Sample demographics	Diagnostic criteria	Main outcome
			Exp	Con			
(19)	China	Case–control study	1,000	1,000	Age, 43.2 years; 36.1% male	Guidelines of Chinese Medical Association	IL1RL1 gene rs72823628, rs950881 and rs3771175 were associated with a reduced AR risk
(20)	Australia	Cross-sectional study	45	24	Age, 37 years; 59% male	ARIA guidelines	AR group showed upregulation of allergy-related genes in nasal mucosa, while 113 genes were differentially expressed in peripheral blood samples and 14 genes in nasal lysate samples
(21)	China	Comparative study	60	60	Age, 27.9 years; 45% male	Based on Chinese Society of Allergy Guidelines for Diagnosis and Treatment of Allergic Rhinitis	Lnc-GAS5 has a negative correlation with its targets (miR-21 and miR-140) in AR, and all three are associated with disease risk, symptom severity, and Th1/Th2 imbalance
(22)	China	Case-control study	500	500	Age, 49.2 years; 39% male	Based on Chinese Society of Allergy Guidelines for Diagnosis and Treatment of Allergic Rhinitis	AA genotype of rs1965708 was associated with increased risk of AR
(23)	China	GWAS study	222	237	Age, 23.2 years; 57.7% male	ARIA guidelines	rs754466 in DLG5 decreased the susceptibility to HDM-induced AR
(24)	China	GWAS study	38	31	Not reported	ARIA guidelines	T-B cell interaction, bridged by CD23 expression, is involved AR progression
(25)	Iran	Case-control study	86	86	Age, 26 years; 42.5% male	ARIA guidelines	No significant association was found between the IL-13 rs20541 polymorphism and AR
(26)	China	GWAS study	20	20	Age, 32.2 years; 50% male	Clinical examination	TNF suppresses IL-10 expression in B cells by increasing HDAC11 expression in AR

AR, allergic rhinitis; ARIA, allergic rhinitis and its impact on asthma (18); GWAS, genome wide association study; TNF, tumor necrosis factor; HDM, house dust mite.

**Table 3:** Investigation of Environmental factors in AR: characteristics of included studies.

Author	Country	Study design	Sample size		Sample demographics	Diagnostic criteria	Main outcome
			Exp	Con			
(27)	Malaysia	Cross-sectional study	30	30	Age, 30 years, 30% male	Clinical examination and skin prick test	AR patients have reduced expression of OCLN and CLDN7 in the nasal epithelial barrier, which is associated with urban locations and exposure to second-hand smoke

AR, allergic rhinitis.

### 3.3. Outcomes of included studies for pathogenesis of allergic rhinitis and non-allergic rhinitis

Due to the heterogeneity in the outcomes of the selected studies, we divided the analysis into three subheadings to ensure a comprehensive evaluation as.

#### 3.3.1. Role of immune mediators in pathogenesis of allergic rhinitis

While various immune mediators have been reported to play a role in the pathogenesis of AR, it is not feasible to cover all of them in a single study. Though we mainly focused on essential mediators of cytokines, along with subtypes for our analysis. However, we also considered other relevant mediators in our study if considered important. We identified five studies from 2018 to 2023 that focused on these immune mediators in the pathogenesis of AR.

The outcomes of these five studies on the pathogenesis of AR indicate that patients with AR had higher serum levels of CCL3, RvE1, and LTB4, but a lower ratio of RvE1 and RvD1 to LTB4, IL-10, and TGF-β cytokines. Additionally, AR patients had more CD4+ T cells and higher levels of IL-10, IL-6, and IFN-γ, but lower levels of Th1 and Th17 subsets with chemokine receptors. There were no significant differences in IL-25 and IL-33 levels between fungal and non-fungal AR. Furthermore, IL-17, IL-22, and TGF-β levels were higher in patients with AR, while IFN-γ levels were lower compared to the control group (13–17). The outcomes of our selected studies regarding the role of immune mediators in pathogenesis of AR are presented in Table 1.

#### 3.3.2. Role of genetic factors in the pathogenesis of allergic rhinitis

There were ten studies that focused on the genetic pathogenesis of AR. Due to the wide range of genetic and epigenetic factors studied, it was not possible to draw a single conclusion for any particular factor in the pathogenesis of AR. The research showed a link between the IL1RL1 gene markers rs72823628, rs950881, and rs3771175, and a lower likelihood of AR. In the AR group, genes connected to allergies were more active in nasal tissue, with 113 genes exhibiting differential expression in peripheral blood samples and 14 genes in nasal lysate samples. Long non-coding RNA GAS5 (lnc-GAS5) showed an inverse relationship with its targets (miR-21 and miR-140) in AR, all of which correlated with disease risk, symptom severity, and Th1/Th2 imbalance. The AA genotype of rs1965708 was associated with a heightened AR risk. The expression of CST1, TFF3, ITLN1, and rs754466 in DLG5 were linked to lower susceptibility to house dust mite-induced AR. T-B cell interaction, bridged by CD23 expression, was involved in AR progression. However, no significant association was found between the IL-13 rs20541 polymorphism and AR (19–26). The outcomes of our selected studies regarding the role of genetic factors in the pathogenesis of AR are presented in Table 2.

#### 3.3.3. Role of environmental factors in pathogenesis of allergic rhinitis

We identified only one study that examined the impact of environmental factors in relation to AR. This study assessed the mRNA expression levels of various tight junctions, including occludin (OCLN), claudin-3 and −7 (CLDN3 and CLDN7), desmoglein 3 (DSG3), and thymic stromal lymphopoietin (TSLP) in 30 individuals with Allergic Rhinitis (AR) and 30 non-allergic controls. The research found a significantly lower expression of OCLN, CLDN3, and CLDN7 transcripts in AR sufferers compared to the non-allergic control group. However, no noticeable variances were seen in the DSG3 or TSLP transcript expression between the two groups. The study also revealed an association between urban areas and decreased OCLN expression, and a correlation between second-hand smoke exposure and lower CLDN7 expression in AR patients. Ultimately, the conclusion was drawn that decreased expression of OCLN and CLDN7, connected to urban settings and second-hand smoke exposure, may be contributing to the impaired nasal epithelial barrier seen in AR patients, supporting recent studies positing air pollution as a possible cause of AR (28).

## 4. Discussion

Chronic rhinitis, and AR in particular, is a major global health concern that affects approximately 400 million people worldwide. The prevalence of AR has been on the rise due to increased urbanization and exposure to environmental pollutants, which are believed to be among the leading causes of the disease. Developing effective therapies for chronic rhinitis requires a thorough understanding of its complex pathophysiology, including various contributing factors (1). Therefore, it is essential to review the complex pathogenesis of AR, considering multiple factors to identify potential drug targets for successful treatment.

Although literature reviews on AR are available, many of them are focused on either single pathology or based on a single pathogenic factor only (29, 30, 31). Our systematic review, on the other hand, takes a comprehensive approach to understand the pathogenesis of AR by considering multiple factors. This is one of the first reviews to adopt such a comprehensive approach. Our selected studies exhibit heterogeneity; therefore, a meta-analysis could not be performed. However, the broad overview of multiple factors in the pathogenesis of chronic rhinitis offers detailed information for researchers to understand its complex mechanism.

In our study, we have identified several cytokines that were reported in the included studies for their involvement in the pathogenesis of AR. The findings from the studies indicate that individuals with AR tend to have elevated levels of certain immune mediators such as CCL3, RvE1, LTB4, CD4+ T cells, IL-10, IL-6, IL-17, IL-22, and TGF-β, but reduced levels of the RvE1 and RvD1 ratio to LTB4, Th1 and Th17 subsets with chemokine receptors, and IFN-γ (13–17).

The role of Cytokines and Chemokines was described in various previous studies. AR is categorized as a type 2 inflammation mediated disease. The pathology of AR involves interleukins 4, 5, and 13, which play significant roles (32). The type 2 inflammatory response is provoked by both the innate immune system, which is activated by pollutants or viral or fungal infections and involves type 2 innate lymphoid cells (ILC2), and the adaptive immune system, which is stimulated by allergen exposure and involves type 2 T-helper (Th2) cells. Both ILC2 and Th2 cells release type-2 cytokines like interleukin IL-4, IL-5, and IL-13, playing diverse roles in the inflammatory cascade. IL-4 and IL-13 cause B-cell class switching and IgE production, provoke the discharge of pro-inflammatory substances, disrupt barriers, and encourage tissue remodeling. In addition, IL-13 prompts an increase in goblet cells leading to excess mucus production (33, 34).

Furthermore, there are indications that the pathophysiology of AR may encompass other pathways, specifically involving the regulation of Th2 cytokine responses by cytokines expressed in the epithelial cells. These cytokines include thymic stromal lymphopoietin, IL-25, and IL-33 (33). In addition to IL-4, IL-5, IL-13, IL-25, and IL-33, a range of other immune mediators contribute to the pathophysiology of AR. In the case of viral infections, elevated levels of various inflammatory cytokines, including IL-1β, IL-6, IL-7, IL-17, IFN-γ, IL-8, TNFα, and GM-CSF are observed, alongside IL-4 and IL-5. Similarly, during the allergy season, the levels of IL-4 and IL-10 decrease in nasal fluid. However, both cytokines demonstrate an increase in concentration 5 h after a nasal allergen challenge conducted outside the allergy season (35). We have also found a decrease in Th17 levels, which is in line with a previous review conducted by Fan et al., suggesting a negative correlation between Th17 and the occurrence of AR. The proposed mechanism is that regulatory B cells play a role in inhibiting the differentiation and proliferation of primary T cells into Th17 cells by facilitating the release of IL-10 and IL-35. As a result, this leads to a reduction in Th17 cells, a decrease in IL-17 secretion, and the alleviation of symptoms in individuals with AR (36).

Chemokine (C-C motif) ligand 3 (CCL3), also known as macrophage inflammatory protein 1-alpha (MIP-1-alpha), is a protein that in humans is encoded by the CCL3 gene and plays a role in inflammatory responses through binding to the receptors CCR1, CCR4 and CCR5 (37). CCL3 stimulates the movement of monocytes and T lymphocytes, although its impact varies among different T cell subsets. For instance, CCL3 primarily drives chemotaxis in B lymphocytes and activated CD8 T cells. This cytokine also prompts the chemotaxis of natural killer (NK) cells. Moreover, CCL3 acts as a chemoattractant for eosinophils and encourages basophils to discharge histamine. Additionally, CCL3 triggers the expression of ICAM-1, the degranulation of mast cells, and the production of tumor necrosis factor alpha (TNF-a), IL-1, and IL-6 (38). In their 2020 study, Berghi and colleagues discovered a notable elevation in the average serum CCL3 levels among patients suffering from Allergic Rhinitis (AR), compared to those in control groups. This finding can be associated with the significant function that CCL3 plays in sparking inflammation in cases of AR.

Endogenous lipid mediators known as Resolvins, specifically Resolvin E1 (RvE1) and Resolvin D1 (RvD1), are identified for their positive influence on resolving inflammation (39). RvE1 has the capability to bind to BLT-1, a receptor with a high affinity for leukotriene B4 (LTB4), which results in the suppression of LTB4-driven immune cell recruitment to inflamed areas. This binding action mitigates the LTB4-induced mobilization of immune cells to inflammation sites, indicating a competition between RvE1 and LTB4 for BLT-1 binding, with RvE1 functioning as a BLT-1 antagonist (40). In a 2020 study conducted by Lotfi and his team, they found that the lower ratios of RvE1 and RvD1 to LTB4 suggest an imbalance in the production of these mediators in AR patients. They suggested that the findings indicate a possibility that LTB4 might be dominating RvE1 in terms of BLT-1 binding, leading to impaired resolution of airway inflammation, and consequently contributing to the development of chronic airway inflammation. Overall, it appears that the increase in RvE1 is a critical action of the immune system to actively resolve allergic airway inflammation, though this effort remains insufficient. Notably, the study also found that serum levels of both IL-10 and TGF-β were significantly decreased in AR patients compared to healthy individuals, which is comparable to findings from a murine study conducted by Oner and associates in (41), where they demonstrated that RvE1 inhibited IL-10 synthesis by Th17 cells. Conversely, their study also revealed that RvE1 increased the production of TGF-β by dendritic cells.

Similar to the focus on cytokines, most of our studies also concentrated on examining the role of genetic factors, specifically in AR. The findings indicated an increase in the expression of allergy-related genes in the nasal mucosa, CD23 expression during T-B cell interaction, and TNF-induced HDAC11 expression. However, the IL1RL1 gene variants, namely rs72823628, rs950881, and rs3771175, were associated with a lower risk of developing AR (19–26). A review by Choi et al. reported multiple genetic and epigenetic factors, some of which were consistent with our findings (29). Lastly, regarding environmental factors, the selected study observed the effect of environmental factors in pathogenesis, and demonstrated an indirect relation between the expression of OCLN and CLDN7 to air pollution (27).

Our review has some limitations that should be addressed in future research. Firstly, the studies included in our review varied in terms of their objectives, methodology, and findings. Similarly, the studies focused on different sub-factors, making it difficult to compare their results. Therefore, future studies should focus on one aspect at a time to obtain more conclusive results. Furthermore, based on our findings, researchers should link these mechanisms to potential therapies for AR and mixed rhinitis.

## 5. Conclusion

Various factors, including immune mediators particularly cytokines, genetic, and environmental factors, are involved in the development of AR. However, the studies presented findings on different factors and sub-factors, making it challenging for us to compare their results. Therefore, future studies should focus on one aspect at a time to obtain more conclusive results. Additionally, researchers should connect these mechanisms to potential treatments for AR and mixed rhinitis based on our findings.

## Data Availability

The original contributions presented in the study are included in the article/Supplementary Material, further inquiries can be directed to the corresponding author.
